# Machine learning prediction model for post- hepatectomy liver failure in hepatocellular carcinoma: A multicenter study

**DOI:** 10.3389/fonc.2022.986867

**Published:** 2022-11-02

**Authors:** Jitao Wang, Tianlei Zheng, Yong Liao, Shi Geng, Jinlong Li, Zhanguo Zhang, Dong Shang, Chengyu Liu, Peng Yu, Yifei Huang, Chuan Liu, Yanna Liu, Shanghao Liu, Mingguang Wang, Dengxiang Liu, Hongrui Miao, Shuang Li, Biao Zhang, Anliang Huang, Yewei Zhang, Xiaolong Qi, Shubo Chen

**Affiliations:** ^1^ Xingtai Key Laboratory of Precision Medicine for Liver Cirrhosis and Portal Hypertension, Xingtai People’s Hospital, Xingtai, Hebei, China; ^2^ Center of Portal Hypertension, Department of Radiology, Zhongda Hospital, Medical School, Southeast University, Nanjing, Jiangsu, China; ^3^ Artificial Intelligence Unit, Department of Medical Equipment Management, Affiliated Hospital of Xuzhou Medical University, Xuzhou, Jiangsu, China; ^4^ School of Information and Control Engineering, China University of Mining and Technology, Xuzhou, Jiangsu, China; ^5^ Department of Hepatobiliary Surgery, Tongji Hospital Affiliated to Huazhong University of Science and Technology, Wuhan, Hubei, China; ^6^ Department of Hepatobiliary Surgery, The First Affiliated Hospital of Dalian Medical University, Dalian, Liaoning, China; ^7^ Department of Hepatobiliary Surgery, Fifth Medical Center of People's Liberation Army (PLA) General Hospital, Beijing, China; ^8^ Institute of Portal Hypertension, The First Hospital of Lanzhou University, Lanzhou, China; ^9^ Department of Microbiology and Infectious Disease Center, School of Basic Medical Sciences, Peking University Health Science Center, Beijing, China; ^10^ Department of Hepatobiliary Surgery, The Second Affiliated Hospital of Nanjing Medical University, Nanjing, Jiangsu, China

**Keywords:** hepatocellular carcinoma, liver resection, post-hepatectomy liver failure, artificial intelligence, machine learning

## Abstract

**Introduction:**

Post-hepatectomy liver failure (PHLF) is one of the most serious complications and causes of death in patients with hepatocellular carcinoma (HCC) after hepatectomy. This study aimed to develop a novel machine learning (ML) model based on the light gradient boosting machines (LightGBM) algorithm for predicting PHLF.

**Methods:**

A total of 875 patients with HCC who underwent hepatectomy were randomized into a training cohort (n=612), a validation cohort (n=88), and a testing cohort (n=175). Shapley additive explanation (SHAP) was performed to determine the importance of individual variables. By combining these independent risk factors, an ML model for predicting PHLF was established. The area under the receiver operating characteristic curve (AUC), sensitivity, specificity, positive predictive value, negative predictive value, and decision curve analyses (DCA) were used to evaluate the accuracy of the ML model and compare it to that of other noninvasive models.

**Results:**

The AUCs of the ML model for predicting PHLF in the training cohort, validation cohort, and testing cohort were 0.944, 0.870, and 0.822, respectively. The ML model had a higher AUC for predicting PHLF than did other non-invasive models. The ML model for predicting PHLF was found to be more valuable than other noninvasive models.

**Conclusion:**

A novel ML model for the prediction of PHLF using common clinical parameters was constructed and validated. The novel ML model performed better than did existing noninvasive models for the prediction of PHLF.

## Introduction

In 2020, primary liver cancer was the sixth most commonly diagnosed cancer and the third leading cause of cancer-related deaths worldwide, as approximately 906,000 new cases and 830,000 deaths occurred in 2020 (1). More than 50% of the world’s total new cases of liver cancer each year are attributed to hepatitis B, which has a high incidence in China (2). Radical liver resection remains the first choice of treatment for hepatocellular carcinoma (HCC) (3). Post-hepatectomy liver failure (PHLF) is the most common cause of postoperative death among patients who undergo hepatectomy for HCC (4). The incidence of PHLF has been reported to be 1.2%-32% and is attributed to different etiologies and surgical procedures (5, 6) as the most common cause of early death after liver surgery (7).

A variety of comprehensive scoring systems and nomogram prediction models can be used to help predict PHLF in patients with HCC (8–10). However, no universally recognized method for the prediction of PHLF has been established. Machine learning (ML), one of the most important branches of artificial intelligence (AI), has undergone rapid development and is being widely used in the field of disease prediction, where it has achieved remarkable results in clinical practice (11). ML is widely used in cancer research, where it is applied to clinical data, radiomics, and genomics to develop predictive models for efficient and accurate decision making (12–14). ML uses computational algorithms to learn from and analyze large amounts of data in a short period of time. Therefore, ML may outperform traditional risk stratification tools *via* the integration of different algorithms such as decision trees, artificial neural networks, random forests, support vector machines, extreme gradient boosting, and light gradient boosting machines (LightGBM) (15). LightGBM uses a histogram-based decision tree algorithm. Compared with other ML models, the LightGBM model is characterized by fast training speed and low memory usage. ML based on LightGBM has only recently been introduced in research involving liver disease (16–18), and the LightGBM model has not yet been used to predict PHLF.

In this study, a novel ML model based on the LightGBM algorithm, namely ML PHLF, was constructed. This novel model may replace traditional scoring systems and facilitate the assessment of liver function and reduction of the incidence of PHLF and postoperative mortality after radical hepatectomy.

## Materials and methods

### Study population

This retrospective study was performed using a multicenter database of patients who underwent radical hepatectomy for HCC at the following hospitals: The Xingtai People’s Hospital, The Second Affiliated Hospital of Nanjing Medical University, Fifth Medical Center of People's Liberation Army (PLA) General Hospital, The First Affiliated Hospital of Dalian Medical University, and Tongji Hospital Affiliated to Huazhong University of Science and Technology. Two independent investigators (JW and JL) reviewed the baseline data, laboratory parameters, treatment records, and pathological findings. All patients were randomly divided into the training, validation, and testing cohorts at a ratio of 7:2:1. This study was performed in accordance with the ethical guidelines of the Declaration of Helsinki and approved by the Institutional Review Board (2022–006).

Patients aged > 18 years with confirmed HCC based on histopathological examination of the tumor specimen and no history of anticancer therapy, including transarterial chemoembolization, ablation, or targeted drugs, were included in this study. Patients who underwent other surgical procedures at the time of hepatectomy and those with insufficient data on important indicators, such as total bilirubin (TBIL) and international normalized ratio (INR) on or after the fifth postoperative day, were excluded from the study.

### Data collection

Patient demographic data, including age, weight, body mass index (BMI), sex, presence of hypertension, etiology of liver disease, and cirrhosis, were retrieved from the medical records. Data on the surgical method (open or minimally invasive), extent of liver resection (major resection: ≥ 3 segments; minor resection:< 3 segments), requirement of intraoperative blood transfusion, number of tumors, maximum tumor diameter, and intraoperative blood loss were extracted from the preoperative and surgical records. Laboratory indicators included red blood cell (RBC) count, white blood cell (WBC) count, platelet (PLT) count, TBIL, direct bilirubin (DBIL), albumin (ALB), alanine aminotransferase (ALT), aspartate aminotransferase (AST), serum alpha-fetoprotein (AFP), carcinoembryonic antigen (CEA), creatinine (Cr), prothrombin time (PT), and INR. Portal hypertension was defined as the presence of varicose veins or a PLT count<100 x 10^9^/L and a spleen diameter > 12 cm. According to previous literature, the model for end-stage liver disease (MELD) score (19) was calculated as:


11.2×ln (INR) +9.57×ln (Cr, mg/dL)+3.78×ln (TBIL, mg/dL)+6.43

The fibrosis-4 (FIB-4) index (20) was calculated as:


AST (U/L)×age (years)/[platelet count (×10^9/L)×alanine aminotransferase ALT (U/L) 1/2]

The albumin-bilirubin (ALBI) score (21) was calculated as:


0.66×lg (TBIL, µmol/L)–0.085×(ALB, g/L)

The aminotransferase-to-platelet ratio index (APRI) score (22) was calculated as:


[AST level (/ULN)/platelet counts (10^9/L)] ×100

Finally, the Child-Turcotte-Pugh (CTP) score (23) was calculated and obtained. Based on the Chinese Society of Hepatology guidelines for the diagnosis and treatment of liver cirrhosis, the diagnosis of liver cirrhosis was made using preoperative clinical variables such as the etiology, history, clinical manifestations, complications, laboratory results, imaging examinations, or liver biopsy histology (24).

### Definition of PHLF

The International Study Group of Liver Surgery (ISGLS) diagnostic criteria for PHLF were used in this study (5). PHLF was defined as an increase in TBIL and INR on or after the fifth postoperative day when compared to preoperative levels, after the exclusion of biliary obstruction as a cause for increased TBIL or INR.

### Development of the ML PHLF model

**Figure 1:**
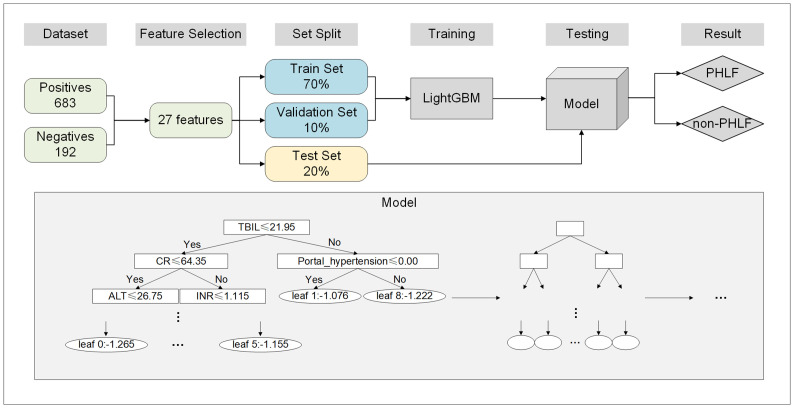
Flowchart of the development of the ML model. ML, machine earning; PHLF, post-hepatectomy liver failure.

A total of 835 patients, including 192 with PHLF and 683 without PHLF, were included in this study. Twenty-five clinical variables, including sex, age, weight, liver disease etiology, cirrhosis, portal hypertension, PLT count, RBC count, WBC count, TBIL, ALB, AST, ALT, DBIL, Cr, PT, INR, AFP, CEA, tumor size, tumor number, surgical approach, extent of resection, intraoperative blood loss, and intraoperative blood transfusion, were used in this study. To verify the performance of the model, 70% of the dataset was used as the training set, 10% was used as the validation set, and 20% was used as the testing set. Data from the training and validation sets were applied to LightGBM, which computed the value of each variable using a decision tree to generate a prediction model for PHLF (**Figure 1**).

The Shapley additive explanation (SHAP), a game-theoretic approach to interpreting the output of the ML PHLF (25, 26), was used to quantitatively measure the importance of each variable and describe the overall relationship between PHLF and all variables. To obtain the best ML model for PHLF, the LightGBM algorithm was optimized by adjusting the number of iterations, number of leaves, and maximum depth of the tree. The optimal number of trees, maximum tree depth, and number of leaves obtained were combined with the hyperparameters adjusted by the validation set to construct an optimal LightGBM model. In addition, the LightGBM algorithm can speed up the training process without affecting the performance of the model. This overall increase in speed is the result of a combination of gradient-based one-sided sampling and exclusive feature bundling. Subsequently, the LightGBM model is used to establish an accurate PHLF diagnosis model with a favorable area under the receiver operating characteristic curve (AUROC). The AUROCs of the training, verification, and testing cohorts were determined.

### Statistical analysis

Continuous variables with normal distribution are presented as median and interquartile range or mean and standard deviation. These variables were compared using Student’s t-test. Non-normal variables were analyzed using the Mann–Whitney rank sum test. Categorical variables are presented as numbers and frequencies (%). The chi-squared test or Fisher’s exact test were used to analyze categorical variables. The predictive performance of the ML PHLF model was assessed using AUROC, sensitivity, specificity, positive predictive value, and negative predictive value (NPV). Decision curve analyses (DCA) were used to measure the clinical utility of each model by calculating the net benefit at various threshold probabilities. R software (version 4.1.2) or Python software (version 3.7.9) was used for data analysis and model building. Statistical significance was set at P< 0.05.

## Results

### Study population

A total of 875 patients were enrolled in this study and randomly assigned to the training (n=612), validation (n=88), and testing (n=175) cohorts at a ratio of 7:1:2. The baseline characteristics of the three groups were not significantly different (**Table 1**).

**Table 1:** Baseline characteristics.

Characteristics	All patients (n=875)	Training cohort (n=612)	Validation cohort (n=88)	Testing cohort (n=175)	P value
PHLF					0.522
No	683 (78.06)	475 (77.61)	73 (82.95)	135 (77.14)	
Yes	192 (21.94)	137 (22.39)	15 (17.05)	40 (22.86)	
Age, years	53 (46 - 60)	54 (46 - 60.2)	53 (45 - 59.2)	52 (46 - 60)	0.967
Weight, kg	67 (60 - 75)	66.5 (60 - 75)	68 (62 - 75.2)	67 (60 - 76.5)	0.268
BMI, kg/m^2^	23.4 (21.5 – 25.4)	23.1 (21.2 – 24.8)	23.7 (22.0 – 25.7)	23.8 (22.0 – 26.1)	0.079
Sex					0.843
Female	140 (16)	99 (16.18)	12 (13.64)	29 (16.57)	
Male	735 (84)	513 (83.82)	76 (86.36)	146 (83.43)	
Cirrhosis					0.796
No	107 (12.23)	78 (12.75)	9 (10.23)	20 (11.43)	
Yes	768 (87.77)	534 (87.25)	79 (89.77)	155 (88.57)	
Portal hypertension					0.961
No	565 (64.57)	395 (64.54)	58 (65.91)	112 (64)	
Yes	310 (35.43)	217 (35.46)	30 (34.09)	63 (36)	
Hepatitis B					0.805
No	105 (12)	73 (11.93)	9 (10.23)	23 (13.14)	
Yes	770 (88)	539 (88.07)	79 (89.77)	152 (86.86)	
Hepatitis C					0.145
No	846 (96.7)	590 (96.4)	88 (100)	168 (96.0)	
Yes	29 (3.3)	22 (3.6)	0 (0)	8 (4.0)	
Alcoholic					0.455
No	870 (99.4)	609 (99.5)	88 (100)	173 (98.9)	
Yes	5 (0.6)	3 (0.5)	0 (0)	2 (1.1)	
Surgical approach					0.668
Laparoscopy	344 (39.31)	235 (38.4)	37 (42.05)	72 (41.14)	
Open surgery	531 (60.69)	377 (61.6)	51 (57.95)	103 (58.86)	
Extent of resection					0.539
Minor	786 (89.83)	545 (89.05)	80 (90.91)	161 (92)	
Major	89 (10.17)	67 (10.95)	8 (9.09)	14 (8)	
Intraoperative blood loss, ml	200 (100 - 482)	200 (100 - 450)	255 (100 - 400)	250 (100 - 500)	0.359
Transfusion					0.069
No	688 (78.63)	494 (80.72)	65 (73.86)	129 (73.71)	
Yes	187 (21.37)	118 (19.28)	23 (26.14)	46 (26.29)	
Tumor number					0.597
Single	744 (85.03)	524 (85.62)	72 (81.82)	148 (84.57)	
Multiple	131 (14.97)	88 (14.38)	16 (18.18)	27 (15.43)	
Tumor size, cm	4 (2.5 - 5)	4 (2.5 - 5.5)	3.2 (2.1 - 5)	4 (2.5 - 5)	0.824
ALT, U/L	31 (22 - 45)	31 (22 - 45)	30.5 (22 - 43.2)	34 (22 - 45)	0.145
AST, U/L	30 (23 - 43)	30 (23 - 43)	29 (24 - 43)	30 (23 - 44)	0.193
Albumin, g/L	39.5 (37 - 42.1)	39.5 (37 - 42.2)	38.8 (36.5 - 42.1)	40 (36.4 - 42)	0.300
Total bilirubin, μmol/L	14 (10.7 - 19)	14.1 (10.8 - 18.9)	14.8 (10.7 - 20.4)	13.6 (10.6 - 18.4)	0.523
Direct bilirubin, μmol/L	4.9 (3.6 - 6.7)	4.9 (3.4 - 6.5)	5.2 (3.7 - 7.3)	4.9 (3.7 - 6.5)	0.256
Creatinine, μmol/L	76 (64.8 - 83)	76 (65 - 84)	75.5 (69 - 81.2)	75 (62.5 - 82.5)	0.718
PT, s	12.3 (11.4 - 13.6)	12.3 (11.4 - 13.5)	12.9 (11.5 - 14)	12.4 (11.5 - 13.6)	0.496
INR	1.1 (1 - 1.1)	1.1 (1 - 1.1)	1.1 (1 - 1.2)	1.1 (1 - 1.1)	0.527
Platelet count, x 10^9^/L	140 (99 - 190)	140 (101 - 188.2)	138.5 (95.2 - 197)	139 (93 - 193)	0.225
RBC count, x 10^12^/L	4.5 (4.2 - 4.9)	4.5 (4.2 - 4.9)	4.5 (4.2 - 4.8)	4.5 (4.1 - 4.8)	0.545
WBC count, x 10^9^/L	5.2 (4.1 - 6.5)	5.1 (4.1 - 6.5)	5 (4.1 - 6.4)	5.3 (4.2 - 6.8)	0.675
AFP, ng/ml	17 (5.4 - 248.7)	18 (5.2 - 286)	17.5 (9.2 - 432.6)	16.6 (5.2 - 166.5)	0.505
CEA, ng/ml	3.1 (1.8 - 3.5)	3 (1.8 - 3.5)	3 (1.9 - 3.5)	3.5 (1.9 - 3.5)	0.462
Machine learning model	0.5 (0.4 - 0.5)	0.5 (0.4 - 0.5)	0.5 (0.4 - 0.5)	0.5 (0.4 - 0.6)	0.564
ALBI	-2.6 (-2.9 - -2.4)	-2.6 (-2.9 - -2.4)	-2.5 (-2.9 - -2.3)	-2.6 (-2.9 - -2.4)	0.490
FIB-4	2.2 (1.4 - 3.5)	2.2 (1.4 - 3.5)	2.1 (1.3 - 3.3)	2.2 (1.3 - 3.7)	0.777
APRI	0.6 (0.4 - 1)	0.6 (0.4 - 1)	0.6 (0.4 - 1)	0.6 (0.3 - 1.1)	0.721
MELD	4.9 (2.9 - 6.7)	4.7 (2.9 - 6.7)	5.3 (3.3 - 7)	4.9 (2.7 - 6.5)	0.611
CTP					0.296
Class A	827 (94.51)	583 (95.26)	82 (93.18)	162 (92.57)	
Class B	48 (5.49)	29 (4.74)	6 (6.82)	13 (7.43)	

Data are presented as number (percentage) or median (range). PHLF, post hepatectomy liver failure; BMI, body mass index; ALT, alanine transaminase; AST, aspartate transaminase; PT, prothrombin time; INR, international normalized ratio; RBC, red blood cell; WBC, write blood cell; AFP, a-fetoprotein level; CEA, carcinoembryonic antigen; ALBI, albumin-bilirubin grade; FIB-4, fibrosis 4 score; APRI, aspartate transaminase to platelet ratio; MELD, model for end-stage liver disease; CTP, Child-Turcotte-Pugh.

### Interpretation of the model using the SHAP algorithm

**Figure 2:**
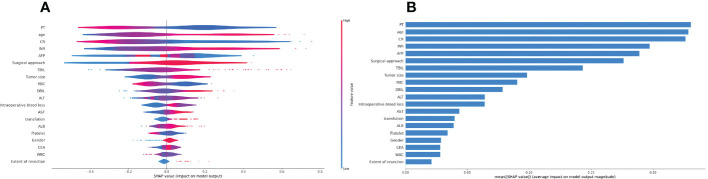
Summary Shapley additive explanations plot revealing the impact of individual clinical variables. **(A)** The importance matrix plot of clinical variables is derived using the LightGBM model. The matrix plot ranks the importance of the variables selected for the final analysis, revealing the contribution of each variable to PHLF versus non-PHLF. **(B)** The SHAP summary plot of the LightGBM model is shown. The higher the SHAP value for each clinical variable, the higher risk of PHLF. LightGBM, light gradient boosting machines; PHLF, post-hepatectomy liver failure.

The top five factors associated with PHLF were PLT count, age, Cr, INR, and AFP (**Figure 2A**). The top 20 variables and the correlation between high or low SHAP values and the predicted PHLF are presented in **Figure 2B**.

### Diagnostic performance of the ML model for PHLF

**Figure 3:**
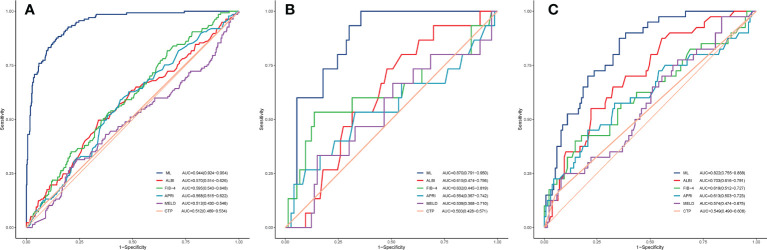
ROC curves. The ROC curves of the FIB-4 score, APRI score, CTP score, MELD score, and ALBI score are compared with that of the ML model in the training **(A)**, validation **(B)**, and testing **(C)** cohorts. ROC, receiver operating characteristic curves; ML, machine learning; FIB-4, fibrosis-4; APRI, aminotransferase to platelet ratio index; CTP, Child-Turcotte-Pugh; MELD, model for end-stage liver disease; ALBI, albumin–bilirubin.

In the training, validation, and testing cohorts, the number of patients with PHLF were 137 (22.4%), 15 (17.1%), and 40 (22.9%), respectively. The areas under the curve (AUCs) of the ML model for detecting PHLF in the training, validation, and testing cohorts were 0.944 (95% confidence interval [CI], 0.924-0.964), 0.870 (95% CI, 0.791-0.950), and 0.822 (95% CI, 0.755-0.888), respectively (**Figure 3**). The ML PHLF model identified PHLF in the training cohort with a sensitivity, specificity, and NPV of 87.6%, 85.9%, and 96.0%, respectively. The sensitivity, specificity, and NPV of the ML PHLF model were 100%, 64.4%, and 100%, respectively, in the validation cohort and 87.5%, 64.4%, and 94.6%, respectively, in the testing cohort (**Tables 2**–**4**).

**Table 2:** Predictive power of the ML model and routine clinical models using data of the training cohort.

Model	Specificity	Sensitivity	Accuracy	NPV	PPV	Recall	Youden’s index
Machine learning model	85.9%	87.6%	86.3%	96.0%	64.2%	87.6%	1.735
ALBI	66.1%	49.6%	62.4%	82.0%	29.7%	49.6%	1.157
FIB-4	33.7%	81.8%	44.4%	86.5%	26.2%	81.8%	1.154
APRI	55.8%	59.1%	56.5%	82.6%	27.8%	59.1%	1.149
MELD	76.2%	31.4%	66.2%	79.4%	27.6%	31.4%	1.076
CTP	95.8%	6.6%	75.8%	78.0%	31.0%	6.6%	1.024

ML, machine learning; NPV, negative predictive value; PPV, positive predictive value; ALBI, albumin-bilirubin grade; FIB-4, fibrosis 4 score; APRI, aspartate transaminase to platelet ratio; MELD, model for end-stage liver disease; CTP, Child-Turcotte-Pugh.

**Table 3:** Predictive power of the ML model and routine clinical models using data of the validation cohort.

Model	Specificity	Sensitivity	Accuracy	NPV	PPV	Recall	Youden’s index
Machine learning model	64.4%	100.0%	70.5%	100.0%	36.6%	100.0%	1.644
ALBI	52.1%	73.3%	55.7%	90.5%	23.9%	73.3%	1.254
FIB-4	86.3%	53.3%	80.7%	90.0%	44.4%	53.3%	1.396
APRI	67.1%	53.3%	64.8%	87.5%	25.0%	53.3%	1.205
MELD	84.9%	33.3%	76.1%	86.1%	31.3%	33.3%	1.183
CTP	0.0%	100.0%	17.0%	–	17.0%	100.0%	1.000

ML, machine learning; NPV, negative predictive value; PPV, positive predictive value; ALBI, albumin-bilirubin grade; FIB-4, fibrosis 4 score; APRI, aspartate transaminase to platelet ratio; MELD, model for end-stage liver disease; CTP, Child-Turcotte-Pugh.

**Table 4:** Predictive power of the ML model and routine clinical models using data of the testing cohort.

Models	Specificity	Sensitivity	Accuracy	NPV	PPV	Recall	Youden’s index
Machine learning model	64.4%	87.5%	69.7%	94.6%	42.2%	87.5%	1.519
ALBI	77.8%	55.0%	72.6%	85.4%	42.3%	55.0%	1.328
FIB-4	85.2%	40.0%	74.9%	82.7%	44.4%	40.0%	1.252
APRI	79.3%	45.0%	71.4%	82.9%	39.1%	45.0%	1.243
MELD	90.4%	25.0%	75.4%	80.3%	43.5%	25.0%	1.154
CTP	94.8%	15.0%	76.6%	79.0%	46.2%	15.0%	1.098

ML, machine learning; NPV, negative predictive value; PPV, positive predictive value; ALBI, albumin-bilirubin grade; FIB-4, fibrosis 4 score; APRI, aspartate transaminase to platelet ratio; MELD, model for end-stage liver disease; CTP, Child-Turcotte-Pugh.

### Comparison of the ML PHLF model and other noninvasive models

We further compared the diagnostic performance of the ML PHLF model with that of routine clinical models, such as ALBI, FIB-4, APRI, MELD, and CTP. The ML PHLF model had the highest AUC for the prediction of PHLF among the noninvasive models (**Figure 3**). In addition, the AUCs for the ALBI, FIB-4, APRI, MELD, and CTP score were 0.570, 0.595, 0.568, 0.512, and 0.512, respectively, in the training cohort (**Figure 3A**); 0.615, 0.632, 0.554, 0.539, and 0.500, respectively, in the validation cohort (**Figure 3B**); and 0.703, 0.619, 0.613, 0.574, and 0.549, respectively, in the testing cohort (**Figure 3C**). The diagnostic performances of routine clinical models in the training, validation, and testing cohorts are summarized in **Tables 2**, **3**, and **4**, respectively.

**Figure 4:**
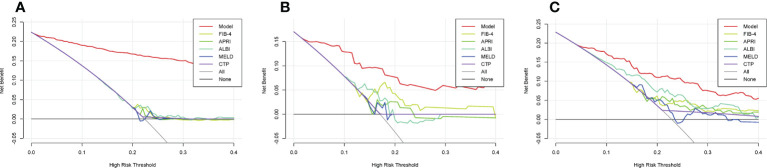
DCA curves. The DCA curves of the FIB-4 score, APRI score, CTP score, MELD score, and ALBI score are compared with that of the ML model in the training **(A)**, validation **(B)**, and testing **(C)** cohorts. sDCA, decision curve analysis; ML, machine learning; FIB-4, fibrosis-4; APRI, aminotransferase-to-platelet ratio index; CTP, Child-Turcotte-Pugh; MELD, model for end-stage liver disease; ALBI, albumin–bilirubin.

The ML PHLF model added more value than did the FIB-4, APRI, ALBI, MELD, or CTP score for predicting PHLF in the training cohort (**Figure 4A**). The results were similar in the validation and testing cohorts. The novel ML PHLF model was more reliable than the traditional models (**Figure 4B** and **Figure 4C**).

### Online calculator application

The ML model is composed of 29 decision trees based on the LightGBM algorithm. Owing to the large number of trees and the complex structure of each tree, only the first three and last two decision trees are shown in **Supplementary Figure S1**. To increase the clinical utility, a web calculator application based on the novel ML model has been developed (http://www.pan-chess.cn/calculator/PHLF_score) (**Supplementary Figure S2**).

## Discussion

In this study, a novel ML PHLF model for predicting the risk of PHLF was developed based on the LightGBM algorithm using the data of 875 patients with HCC who underwent liver resection. This novel model exhibited the best AUC when compared to existing noninvasive prediction models and good decision making in the training, validation, and testing cohorts in this study. This valuable and reliable predictive model for PHLF may be effective in optimizing personalized treatment options for patients with HCC, allowing for early identification of patients with HCC at high risk for PHLF.

Radical liver resection remains the first-choice treatment for HCC. With the update of new surgical techniques, optimization of innovative surgical instruments, and advancement of surgical intensive care medicine, the safety of liver resection has significantly improved. Therefore, perioperative mortality after hepatectomy has decreased (27). However, PHLF remains a serious complication for patients with HCC after hepatectomy. Accurate identification of patients with a high risk of PHLF is critical. Therefore, development of a predictive model for PHLF is crucial for clinical decision making.

Previously reported, noninvasive models for the prediction of PHLF are mainly based on laboratory indicators. The FIB-4, APRI, CTP score, MELD, and ALBI are widely used scoring systems for the evaluation of liver function and have been confirmed to predict the occurrence of PHLF (28–32). However, the predictive efficacy of these traditional noninvasive models that are based on simple laboratory indicators is relatively poor, whereas AI-based combined models using multiple clinical parameters have greater predictive potential.

ML is a field of AI that uses data-driven mining of complex datasets to predict future outcomes (33–35). The use of various ML algorithms to perform disease risk prediction has become a research hotspot in the field of medical big data. Various complex algorithms can be used to deeply mine the relationships between disease variables. The ML model has two advantages over other models, including the use of nonlinear functions and the consideration of the possible effects between all variables. ML algorithms have been increasingly applied to pertinent issues in the field of liver surgery (16). Mai et al. (36) developed an artificial neural network-based model to predict the risk of PHLF in patients with HCC undergoing partial hepatectomy. The predictive performance of the model exceeded that of traditional logistic regression models and commonly-used scoring systems. However, no research regarding ML models developed based on LightGBM that predict PHLF has been reported.

Twenty-five clinically meaningful variables were used to develop the ML PHLF model according to the SHAP analysis in this study. Specifically, both the importance matrix plot and the SHAP results indicate that PLT count, age, Cr, INR, and AFP are the five most important contributors to the final model. The preoperative PLT count was identified as the most important factor. A meta-analysis of 13 studies (37) assessed the effects of perioperative PLT count on PHLF and mortality using two PLT count cutoffs (100 and 150 platelets/nL). Patients with a perioperative PLT count< 150/nL (four studies, 817 patients; odds ratio [OR]: 4.79; 95% CI, 2.89-7.94) and those with a PLT count< 100/nL (four studies, 949 patients; OR: 4.65; 95% CI: 2.608.31) had a high risk of developing PHLF (37). As shown in previous studies, PLT count (38), age (39), Cr (40), INR (41), and AFP (42) are all predictors of PHLF.

The ML PHLF model is more accurate for the prediction of PHLF than are existing models and is convenient to use. The AUCs of the ML PHLF model for detecting PHLF in the training, validation, and testing cohorts were 0.944, 0.870, and 0.822, respectively, confirming that the ML PHLF model has good predictive value for PHLF in different groups. The ML PHLF model had the highest predictive value for AUC among traditional scoring systems in all three cohorts. The ML model identified PHLF in the training cohort with a sensitivity, specificity, and NPV of 87.6%, 85.9%, and 96.0%, respectively. The sensitivity, specificity, and NPV of the ML PHLF model were 100%, 64.4%, and 100%, respectively, in the validation cohort and 87.5%, 64.4%, and 94.6%, respectively, in the testing cohort. When compared to the sensitivity, specificity, and NPV of traditional scoring systems, those of the ML PHLF model were the highest. The ML PHLF model outperformed traditional noninvasive models according to the DCA curves. To facilitate the use of this model in the clinic, a free web calculator to predict the risk of PHLF has been developed (http://www.pan-chess.cn/calculator/PHLF_score).

This is the first multicenter study to explore the development and validation of a LightGBM-based model for the prediction of PHLF in patients with HCC. The ML PHLF model is based on routine clinical parameters obtained in patients with HCC. With the advantages of convenient data collection, availability, and objectiveness, the novel model is suitable for the prediction of PHLF in most clinical situations, showing good interpretability and consistency with clinical experience and demonstrating good reliability.

However, this study has some limitations. First, a selection bias was unavoidable; however, this offset has been minimized *via* the multicenter design. Second, the ML PHLF model is poorly interpretable, a black box, and prone to overfitting. Therefore, interpretable ML algorithms will be assessed in follow-up studies. Last, the novel ML model predicts the overall risk of PHLF as defined by the ISGLS criteria. Prospective multicenter studies are required to determine the predictive value of ML PHLF models in CTP class B and C subgroups and other PHLF diagnostic criteria, such as the 50-50 criteria.

## Conclusion

In conclusion, an ML PHLF model using common clinical parameters was constructed and validated based on the LightGBM algorithm. Compared to other noninvasive models, this novel model has the best PHLF-predictive ability. This model can be used to help accurately predict the risk of PHLF, screen high-risk PHLF subgroups, and help surgeons determine personalized treatment options.

## Data availability statement

The raw data supporting the conclusions of this article will be made available by the authors, without undue reservation.

## Ethics statement

The studies involving human participants were reviewed and approved by Ethics Committee of Xingtai People’s Hospital of Hebei Province. The patients/participants provided their written informed consent to participate in this study.

## Author contributions

All authors contributed to the study conception and design. Material preparation, data collection and analysis were performed by TZ, YoL, SG, JL, ZZ, DS, CheL, PY, YH, ChuL, YaL, ShaL, MW, DL, HM, ShuL, BZ, and AH. The first draft of the manuscript was written by JW and all authors commented on previous versions of the manuscript. All authors read and approved the final manuscript.

## Conflict of interest

The authors declare that the research was conducted in the absence of any commercial or financial relationships that could be construed as a potential conflict of interest.

## Publisher’s note

All claims expressed in this article are solely those of the authors and do not necessarily represent those of their affiliated organizations, or those of the publisher, the editors and the reviewers. Any product that may be evaluated in this article, or claim that may be made by its manufacturer, is not guaranteed or endorsed by the publisher.
