# Corrigendum: Machine learning prediction model for post- hepatectomy liver failure in hepatocellular carcinoma: a multicenter study

**DOI:** 10.3389/fonc.2023.1278295

**Published:** 2023-08-31

**Authors:** Jitao Wang, Tianlei Zheng, Yong Liao, Shi Geng, Jinlong Li, Zhanguo Zhang, Dong Shang, Chengyu Liu, Peng Yu, Yifei Huang, Chuan Liu, Yanna Liu, Shanghao Liu, Mingguang Wang, Dengxiang Liu, Hongrui Miao, Shuang Li, Biao Zhang, Anliang Huang, Yewei Zhang, Xiaolong Qi, Shubo Chen

**Affiliations:** ^1^ Xingtai Key Laboratory of Precision Medicine for Liver Cirrhosis and Portal Hypertension, Xingtai People’s Hospital, Xingtai, Hebei, China; ^2^ Center of Portal Hypertension, Department of Radiology, Zhongda Hospital, Medical School, Southeast University, Nanjing, Jiangsu, China; ^3^ Artificial Intelligence Unit, Department of Medical Equipment Management, Affiliated Hospital of Xuzhou Medical University, Xuzhou, Jiangsu, China; ^4^ School of Information and Control Engineering, China University of Mining and Technology Xuzhou, Jiangsu, China; ^5^ Department of Hepatobiliary Surgery, Tongji Hospital Affiliated to Huazhong University of Science and Technology, Wuhan, Hubei, China; ^6^ Department of Hepatobiliary Surgery, The First Affiliated Hospital of Dalian Medical University, Dalian, Liaoning, China; ^7^ Department of Hepatobiliary Surgery, Fifth Medical Center of PLA General Hospital, Beijing, China; ^8^ Institute of Portal Hypertension, The First Hospital of Lanzhou University, Lanzhou, China; ^9^ Department of Microbiology and Infectious Disease Center, School of Basic Medical Sciences, Peking University Health Science Center, Beijing, China; ^10^ Department of Hepatobiliary Surgery, The Second Affiliated Hospital of Nanjing Medical University, Nanjing, Jiangsu, China

**Keywords:** hepatocellular carcinoma, liver resection, post-hepatectomy liver failure, artificial intelligence, machine learning

In the published article, there were two errors regarding the affiliations for Yifei Huang and Yanna Liu. The affiliation for Yifei Huang was displayed as “Center of Portal Hypertension, Department of Radiology, Zhongda Hospital, Medical School, Southeast University, Nanjing, Jiangsu, China” and for Yanna Liu was displayed as “Center of Portal Hypertension, Department of Radiology, Zhongda Hospital, Medical School, Southeast University, Nanjing, Jiangsu, China”. The correct affiliation for Yifei Huang is “Institute of Portal Hypertension, The First Hospital of Lanzhou University, Lanzhou, China” and for Yanna Liu is “Department of Microbiology and Infectious Disease Center, School of Basic Medical Sciences, Peking University Health Science Center, Beijing, China”.

The authors apologize for this error and state that this does not change the scientific conclusions of the article in any way. The original article has been updated.

