# Supplementary material for: Machine learning prediction model for post- hepatectomy liver failure in hepatocellular carcinoma: A multicenter study
Source: Front Oncol. 2022 Nov 2;12:986867. doi: 10.3389/fonc.2022.986867 (PMC9667038; doi:10.3389/fonc.2022.986867)
Supplement: Supplementary file 1 [file Table_1.docx]

Supplementary Material

## Supplementary Figures

**Supplementary Figure S1.** The whole statistical analysis flow chart of ML model.

Light gradient boosting machines (LightGBM), the core algorithm of our machine learning model, is a gradient boosting framework that uses decision trees as base learners. Its basic idea is to superimpose the base classifiers layer by layer, and give higher weight to the samples misclassified by the previous base classifiers during training for each layer. When testing, the final result is obtained according to the weighting of the results of each layer classifier. Therefore, the model trained by LightGBM is composed of multiple decision trees. In this study, the model is composed of 29 decision trees. Due to the large number of trees and the complex structure of each tree, we show the first three and last two decision trees here.

Abbreviations: ML,Machine Learning; TBIL, total bilirubin; CR, creatinine; ALT, alanine transaminase; INR, international normalized ratio; PT, prothrombin time; RBC, red blood cell; ALB, albumin; AST, aspartate transaminase; AFP, a-fetoprotein level; DBIL, direct bilirubin,

**Supplementary Figure S2.** The online calculators based on our machine learning model for predicting the probability of post-hepatectomy liver failure.

Abbreviations: PHLF, post hepatectomy liver failure.
