# Supplementary figures and images for: Machine learning prediction model for post- hepatectomy liver failure in hepatocellular carcinoma: A multicenter study
Source: Front Oncol. 2022 Nov 2;12:986867. doi: 10.3389/fonc.2022.986867 (PMC9667038; doi:10.3389/fonc.2022.986867)

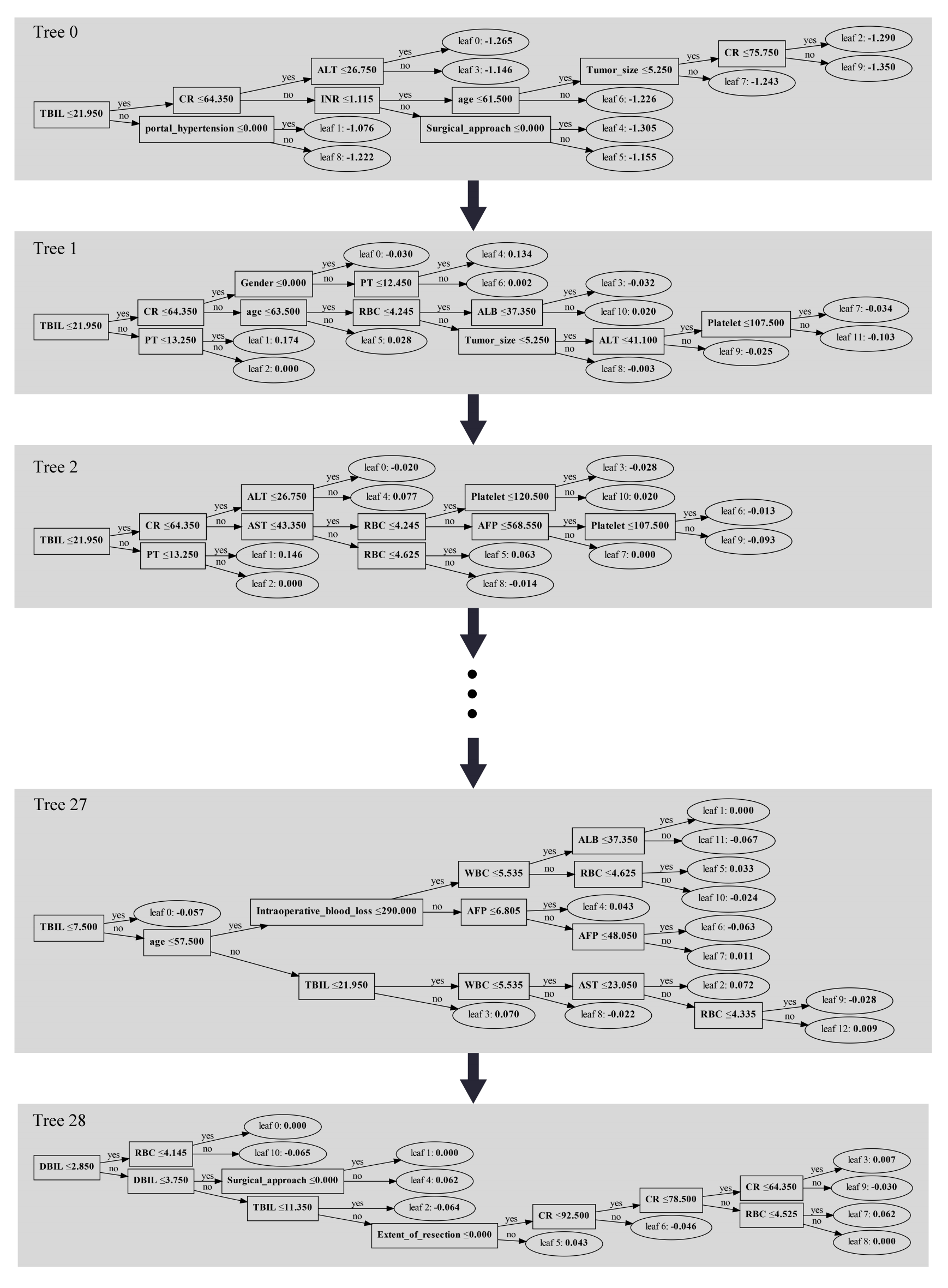

Supplement: Supplementary file 2 [file Image_1.tif]

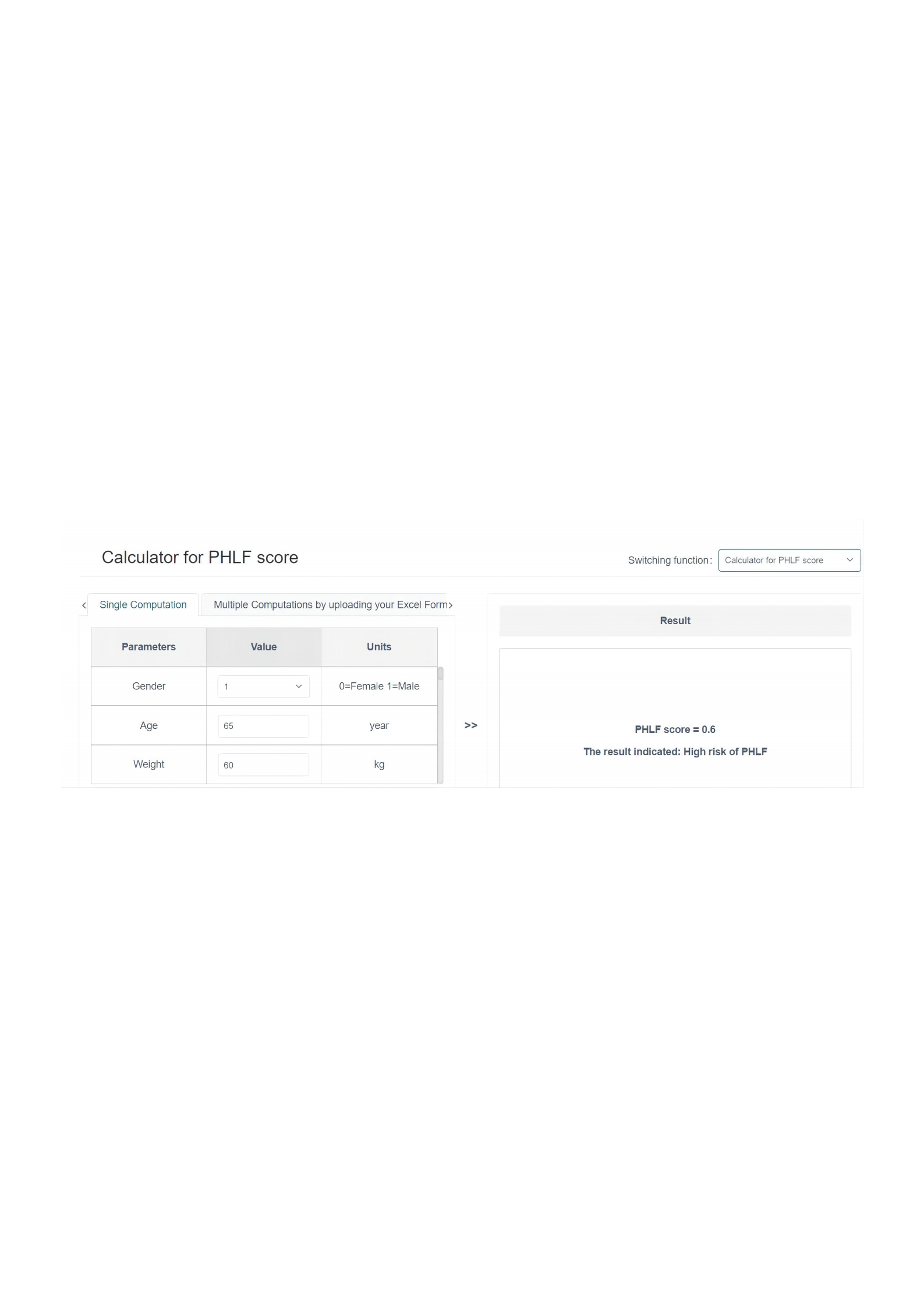

Supplement: Supplementary file 3 [file Image_2.tif]
